# Pressure makes diamonds? The impact of closed incision negative pressure wound therapy on complications following abdominoplasty in patients with massive weight loss—results from a case controlled trial

**DOI:** 10.1016/j.jpra.2024.09.004

**Published:** 2024-09-11

**Authors:** Torsten Schulz, Toralf Kirsten, Katharina Theresa Vogel, Stefan Langer, Rima Nuwayhid

**Affiliations:** a Department of Orthopaedic, Trauma and Plastic Surgery, University Hospital Leipzig, 04103 Leipzig, Germany; b Medical Informatics Center, Department of Medical Data Science, University Hospital Leipzig, 04103 Leipzig, Germany

**Keywords:** Negative pressure wound therapy, Abdominoplasty, Complications, VAC, Seroma, Massive weight loss, Post-bariatric surgery

## Abstract

**Background:**

The effectiveness of closed incision negative pressure wound therapy (ciNPWT) has been shown across various studies. However, studies with large patient cohorts comprising post-bariatric patient populations are missing. The objective of this research was to assess the influence of ciNPWT on post-operative wound complications in this demanding patient collective.

**Methods:**

We conducted a retrospective case-control study. Between 1 January 2013 and 31 December 2023, a total of 251 abdominoplasty procedures following massive weight loss were identified. Patients were matched based on resection weights. We matched 118 patients separated into two groups depending on post-surgical wound management (conventional wound dressings vs ciNPWT). The primary outcomes were wound-related disorders and secondary outcomes were the number of readmissions or reoperations within 30 days after the initial surgery.

**Results:**

The study revealed equal incidence of seroma formation (15 vs 15, p = 1.0), rates of wound dehiscence (23 vs 20, p = 0.56), surgical site infection (11 vs 6, p = 0.18), hematoma (17 vs 9, p = 0.07), complete removal of all drainages (6.7 vs 6.1 days, p = 0.34) and total number of readmission (12 vs 11, p = 0.77) or reoperations (12 vs 10, p = 0.63) within 30 days. The second hospital stay caused by revision was significantly shorter in the ciNPWT group (5.8 days vs 12.0 days, p = 0.02).

**Conclusion:**

Consequently, we did not find evidence to support the hypothesis that ciNPWT reduces complications after abdominoplasty in patients with massive weight loss.

## Introduction

Closed incision negative pressure wound therapy (ciNPWT) is an advancement of conventional negative pressure wound therapy.^1^ Recognised mechanisms of action for ciNPWT include shielding the incision site from external pathogens and aiding in the approximation of incision edges. Furthermore, ciNPWT contributes to the evacuation of fluids and infectious substances from surgical sites, which holds particular significance in critical anatomical regions.^1^ Various clinicians have reported on the positive effects of ciNPWT,^2^, ^3^, ^4^ particularly in preventing the superficial wound breakdown and reducing reoperations due to wound complications.^5^ Several studies on ciNPWT use in plastic surgery have emerged, but only two studies report on the effect of ciNPWT in abdominoplasties within the context of post-bariatric surgery.^4^^,^^6^ Moreover, these studies feature rather small patient cohorts. Therefore, the purpose of this investigation was to determine the effect of ciNPWT in this retrospective case-control study on a larger patient collective. We hypothesised that ciNPWT reduces wound-related complications and revision surgeries.

## Material and Methods

### Patients and Outcomes

This retrospective case-control study was carried out at a single institution with permission from the institutional ethical review board and was created according to the STROBE guidelines. Data were retrieved from the digital patient records of every abdominoplasty performed at the University Hospital Leipzig, covering the period from 1 January 2013 to 31 December 2023. Patient data were prepared using the services of our university hospital's Data Integration Centre, funded by the German Federal Ministry of Education and Research (Grant No 01KX2121). Through a database query, all post-bariatric procedures were recorded anonymously. The cases were included based on the main diagnosis codes. Patients treated in other departments, without massive weight loss and those with incomplete datasets were excluded. The captured data were merged by the Department of Medical Data Science of the Medical Informatics Centre using the statistical software R (RStudio Team 2020, RStudio: Integrated Development Environment for R. RStudio, PBC, Boston, MA, USA). The patients were assigned to two different groups: one received ciNPWT for post-surgical wound management (ciNPWT group), whereas the other was treated conventionally with standard dressings (conventional group). Among the collected variables were age, gender, body mass index (BMI), relevant comorbidities, laboratory data and anesthesiology parameters. Additionally, characteristics of the performed procedure such as cut-stitch time and additional procedures such as liposuction, hernia closure or rectus plication were documented.

### Surgical Techniques and Post-operative Wound Management

Abdominoplasty, a commonly performed surgical procedure, entails making a substantially long incision, typically extending from the left to right anterior superior iliac spine, tracing the natural suprapubic crease. An epifascial flap is then created, extending upwards to the costal margins and reaching the xiphoid process. Particular attention is paid to preserving the umbilicus, leaving it attached to the abdomen via the umbilical stalk and fat pad to maintain adequate blood supply. After lifting the flap, the patient's positioning is adjusted to finalise its placement, mark excess skin and ensure proper closure. Excess skin and fat are subsequently excised, and the flap is carefully reattached to optimise scar healing.^7^ The umbilicus is repositioned on the abdominal wall. This involves anchoring the belly button to the rectus sheet with PDS 3-0 sutures, creating an incision in the form of an inverted V at the new position in the abdominal wall, and then relocating the belly button to its new position over this incision. After resection of the excess tissue, two 12 Ch and two 14 Ch Redon drains are inserted. Wound closure is performed in multiple layers, starting with the Scarpa's fascia using Monocryl 0, followed by the dermis with Monocryl 3-0. The skin is then sutured intradermally continuously using Monocryl 4-0. The newly reinserted umbilicus is sutured to the dermis using Monocryl 3-0 and Ethilon 4-0. Later, the belly button is covered with an antiseptic dressing consisting of Betadine ointment, fat gauze and adhesive tape and the lower abdominal skin incision is either covered with reinforced adhesive skin closures (3M™ Steri-Strip™) or a vacuum dressing. Additional procedures might entail simultaneous liposuction, hernia closure or rectus plication.^7^ Abdominoplasties are a standard procedure in our clinic and is always performed by an attending physician and one or two residents. The technique has remained unchanged over the years, ensuring consistent surgical quality by the attending physician. The decision to use ciNPWT or conventional dressings was made preoperatively by the attending physician in consultation with the patient. The indication for ciNPWT is made when a high resection weight was expected.

CiNPWT dressing is administered using a portable negative pressure wound therapy system (KCI, San Antonio, Texas, USA). CiNPWT has been used since the beginning of the described time period. The vacuum sponge is cut into four horizontal pieces with a scalpel and applied to the wound along with fat gauze to protect the skin. CiNPWT was maintained until post-operative day 7. On the eighth post-operative day, the wound was covered with reinforced adhesive skin closures. In the control group, reinforced adhesive skin closures were applied for wound management until post-operative day 21. Patients were instructed to sustain a flexed (Semi-Fowler) position for 7 days post-operatively. This posture is critical in lessening the tension on the incision and mitigating the potential for hypertrophic scar formation.

### Primary and Secondary Outcome

The primary endpoint encompassed the presence of seromas, wound dehiscence, surgical site infections (SSI), abdominal wall hematomas, timing of drain removal, occurrence of post-operative bleeding, requirement for erythrocyte concentrate transfusion and readmission or surgical revision within 30 days after surgery. Therefore, seroma was classified as an increase in fluid requiring drainage procedures such as aspiration, drain placement or surgical capsulotomy. According to internal clinical standards, drainage removal was conducted at a drainage output of 30 ml/day. Drainage tubes were left in place for a maximum of 10 days before being removed. Wound dehiscence was characterised by the separation of the wound from its margins, either partially or completely. Furthermore, hematoma was identified as a localised accumulation of blood outside the blood vessels within the tissue. Diagnosis of wound infections relied on the clinical signs of inflammation (such as rubor, calor, dolor, tumour and functio laesia) and included cases necessitating operative drainage, microbiological evidence of pathogens and/or antibiotic administration beyond routine post-operative care. Post-operative bleeding was defined as haemorrhage occurring within body cavities or tissues, either externally from incisions requiring surgical interventions or necessitating return to the operating room. Transfusions were performed at serum haemoglobin levels of 4.3 mmol/l or less. Secondary endpoints encompassed the number of patients readmitted to the hospital within 30 days, quantity of operative reinterventions and incidence of revision surgeries.

### Statistical Analysis

The study provided an overview of the patients’ baseline characteristics, presenting means ± standard deviations (SD) for continuous parametric variables and rounded frequencies (%) for categorical variables. Primary and secondary outcomes were compared between the ciNPWT and control groups using appropriate statistical tests, the unpaired t-tests or Chi-squared tests depending on data type. The statistical analyses were performed using IBM SPSS Statistics, Version 29 (IBM Corporation, Armonk, NY, USA). To achieve a balanced distribution of patient characteristics, the resection weights of both groups were matched to eliminate confounding variables. One control was assigned per case. A two– tailed significance level of <0.05 was set to reject the null hypothesis and accept the alternative hypothesis.

## Results

### Patient Cohort, ciNPWT Group vs Conventional Group

**Figure 1:**
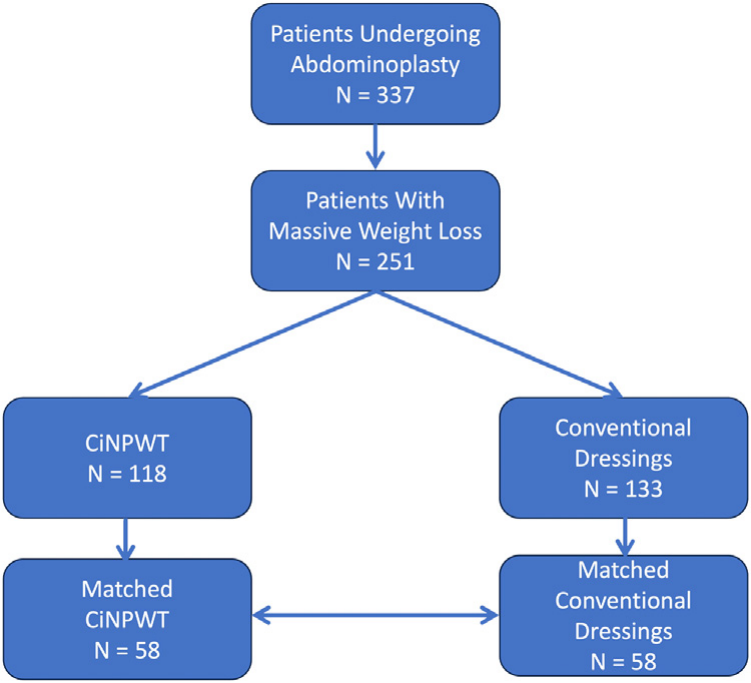
Study protocol.

Between January 2013 and December 2023, a total of 337 patients who underwent abdominoplasty were identified at our clinic. Among these, 251 patients had experienced significant weight loss before, with 118 receiving vacuum dressings. Following the formation of the two groups, matching of resection weights was conducted to ensure comparable cohorts (Figure 1). Ultimately, 58 patients were assigned to each group (Table 1). The average age of both groups was approximately 50 years, with most patients being female. Over ten patients in each group achieved weight reduction through dietary measures, whereas the majority underwent bariatric interventions. Both groups showed no significant differences in baseline BMI (p > 0.85), relative BMI reduction (p = 0.74), BMI before post-bariatric abdominoplasty (p = 0.29), surgical duration (p > 0.56), resection volume (p > 0.74) and time between weight loss and abdominoplasty (p > 0.06). Moreover, both groups exhibited a similar distribution of American Society of Anesthesiologists - status and intraoperative procedures. The only distinction was observed in the length of hospital stay, with 6.8 days in the control group and 8.3 days in the ciNPWT group (p < 0.01), attributed to the 7-day application of the epidermal vacuum dressing as our clinical standard.

**Table 1:** Patient baseline data.

	Conventional (n=58)	CiNPWT (n=58)	p
Male, N/%	22/ 37.9%	11/18.9%	
Female, N/%	36/62.0%	47/81.0%	
Age in years, mean ± SD	49.7 ± 22.0	53.6 ± 10.9	0.23
**Bariatric history**			
post-bariatric procedures, N/%	45/77.5%	47/81.0%	
Weight loss by diet, N/%	13/22.4%	11/18.9%	
BMI, kg/m^2^ initial, mean ± SD	53.4 ± 9.0	53.8 ± 10.2	0.85
BMI, kg/m^2^ reduction, mean ± SD	20.6 ± 9.2	20.0 ± 9.8	0.74
BMI, kg/m^2^ after weight loss, mean ± SD	32.8 ± 4.2	33.7 ± 4.9	0.29
**Post-bariatric metrics**			
Time between bariatric and post-bariatric surgery in days, mean ± SD	1153.5 ± 567.5	1330.2 ± 546.6	0.06
ASA I, N/%	4/6.8%	3/5.1%	0.69
ASA II, N/%	46/79.3%	45/77.5%	0.82
ASA III, N/%	8/13.7%	10/17.2%	0.60
ASA IV, N/%	0/0%	0/0%	1.0
**Additional intraoperative procedures**			
Liposuction, N/%	11/18.9%	11/18.9%	1.0
Lipoaspirate (ml), mean ± SD	2290.0 ± 1447.9	1331.8 ± 740.3	0.08
Rectus plication, N/%	11/18.9%	9/15.5%	0.62
Hernia closure by direct suture, N/%	10/17.2%	10/17.2%	1.0
**Perioperative metrics**			
Cut-to-stitch time, min, mean ± SD	142.8 ± 43.8	147.3 ± 40.3	0.56
Resection weight, g, mean ± SD	3417.7 ± 1436.3	3505.0 ± 1381.7	0.74
Hospital stay, days, mean ± SD	6.8 ± 3.5	8.3 ± 2.5	< 0.01

In both groups, a uniform distribution of pre-existing conditions was observed (Table 2). The most prevalent conditions included arterial hypertension, hypothyroidism, type II diabetes mellitus, chronic nicotine abuse, hepatopathies or venous insufficiencies. The only significant difference was the higher incidence of psychological disease (p < 0.04) in the ciNPWT group. Upon examination of preoperative laboratory data, both groups exhibited normal values for complete blood count, coagulation parameters, electrolytes and HbA1c. No significant differences were observed (Table 3). Further analysis of bariatric laboratory parameters revealed decreased levels of iron, zinc and vitamin B1 in both groups, whereas vitamin B6 levels were significantly elevated. Ferritin, folic acid and vitamin B12 levels were within normal ranges. Concerning this basic blood work, there was no evidence of statistically significant differences between the treatment groups.

**Table 2:** Patient comorbidities.

	Conventional (n=58) N/%	ciNPWT (n=58) N/%	p
Hypertension	31/53.4%	33/56.8%	0.70
Hypothyroidism	18/31.0%	20/34.4%	0.69
Diabetes mellitus type II	17/29.3%	15/25.8%	0.67
Smokers	14/24.1%	10/17.2%	0.35
Hepatopathies	11/18.9%	16/27.5%	0.27
Venous insufficiency	10/17.2%	8/13.7%	0.60
Neurological disease	9/15.5%	12/20.6%	0.46
Rheumatism	6/10.3%	3/5.1%	0.29
Dyslipidaemia	6/10.3%	10/17.2%	0.99
Psychological diseases	5/8.6%	13/22.4%	0.04
Pulmonary diseases	5/8.6%	11/18.9%	0.11
OSAS	4/6.8%	5/8.6%	0.72
Coronary heart disease	3/5.1%	3/5.1%	1.00
Cardiac arrhythmias in the past	3/5.1%	4/6.8%	0.69
Chronic heart failure	3/5.1%	1/1.7%	0.30
Chronic alcohol abuse	3/5.1%	2/3.4%	0.64
Chronic kidney insufficiency	3/5.1%	9/15.5%	0.06
Anaemia	3/5.1%	1/1.7%	0.31
Thromboembolism (previous)	2/3.4%	6/10.3%	0.14
Lipoedema	2/3.4%	3/5.1%	0.64
Multi resistant bacteria	2/3.4%	5/8.6%	0.24
Coagulopathies	1/1.7%	4/6.8%	0.17
Cerebrovascular disease	1/1.7%	2/3.4%	0.55
Hyperthyroidism	0/0%	2/3.4%	0.15
Pneumonia (previous)	0/0%	1/1.7%	0.31

**Table 3:** Preoperative laboratory.

	Unit	Reference Range	Conventional (n=58)	ciNPWT (n=58)	p
Haemoglobin	mmol/l	[8.4-10.9]	8.1 ± 1.1	8.4 ± 0.9	0.22
Mean corpuscular haemoglobin	fmol	[1.7-2]	1.7 ± 0.1	1.8 ± 0.1	0.06
Mean corpuscular volume	fl	[80-96]	85.1 ± 6.4	86.9 ± 4.8	0.10
White blood cells	exp 9/l	[3.5-9.8]	6.7 ± 1.9	7.3 ± 1.9	0.06
Platelets	exp 9/l	[140-360]	244.8 ± 63.3	265.2 ± 79.2	0.13
CRP	mg/l	[< 5]	2.5 ± 3.0	2.7 ± 4.8	0.77
Quick	%	[>70]	101.0 ± 14.3	101.4 ± 10.3	0.86
PTT	s	[25-37]	30.0 ± 3.0	29.2 ± 3.3	0.23
Albumin	mg/l	[<23,8]	43.0 ± 2.7	42.7 ± 6.5	0.78
Ca^2+^	mmol/l	[2.19-2.54]	2.3 ± 0.1	2.3 ± 0.1	0.64
Na+	mmol/l	[135-145]	140.8 ± 1.8	140.5 ± 2.4	0.41
K+	mmol/l	[3.75-5.1]	4.3 ± 0.7	4.5 ± 0.3	0.10
ASAT	µkat/l	[0.17-0.85]	0.4 ± 0.1	0.4 ± 0.1	0.33
ALAT	µkat/l	[0.17-0.85]	0.3 ± 0.1	0.4 ± 0.1	0.21
GFR	ml/min/1.73 m^2^	[<70]	95.1 ± 19.9	88.7 ± 17.1	0.06
HbA1c	%	[<7.5]	5.3 ± 0.6	5.7 ± 1.0	0.08
**Bariatric laboratory, N=92**					
Ferritin	ng/ml	[13-150]	80.2 ± 15.4	93.0 ± 19.0	0.44
Fe2+	µmol/l	[8.8-27]	7.3 ± 1.9	4.7 ± 1.2	0.36
Zink	µmol/l	[11.0-24.0]	2.0 ± 0.4	1.8 ± 0.4	0.09
Folic acid	nmol/l	[8.8-45.4]	10.0 ± 2.1	10.2 ± 2.7	0.67
Vitamin B12	pmol/l	[145-569]	210.69 ± 40.5	177.8 ± 37.9	0.38
Vitamin B1	nmol/l	[66.5-200]	62.7 ± 12.3	48.9 ± 10.2	0.75
Vitamin B6	pmol/l	[35-110]	169.3 ± 34.5	396.1 ± 114.3	0.21

### Primary and Secondary Endpoints

When considering the primary endpoints, the ciNPWT group showed no statistically significant difference in the occurrence of wound dehiscence, SSI and hematomas compared to the control group (Table 4). Further, the incidence of clinically significant seromas was equal in both groups, with 15 subjects each. The drainage removal occurred in the ciNPWT group on day 6.1 compared to day 6.7 in the control group (p = 0.34). Moreover, events such as post-operative bleeding (p = 0.50) or the need for transfusion (p = 0.69) were not statistically significant.

**Table 4:** Primary and secondary outcome parameters.

	Non-NPT (n=58)	CiNPWT (n=58)	p
**Primary Outcome**			
*Minor complications*			
Seroma, N/%	15/25.8%	15/25.8%	1.0
Wound dehiscence, N/%	23/39.6%	20/34.4%	0.56
Surgical site infections, N/%	11/18.9%	6/10.3%	0.18
Hematoma, N/%	17/29.3%	9/15.5%	0.07
Removal of all drains in days, mean ± SD	6.7 ± 1.6	6.1 ± 1.0	0.34
*Major complications*			
Post-operative bleeding, N/%	4/6.8%	6/10.3%	0.50
Transfusion, N/%	3/5.1%	4/6.8%	0.69
**Secondary Outcome**			
Readmission to hospital within 30 days, N/%	12/20.6%	11/18.9%	0.77
Return to operating room within 30 days, N/%	12/20.6%	10/17.2%	0.63
*Number of revision operations*			
One necessary revision, N/%	7/12.0%	9/15.5%	0.38
Two necessary revisions, N/%	4/6.8%	1/1.7%	0.27
Three necessary revisions, N/%	1/1.7%	0/0%	0.31
Hospital stay after revision, days, mean ± SD	12.0 ± 5.6	5.8 ± 3.2	0.02

**Figure 2:**
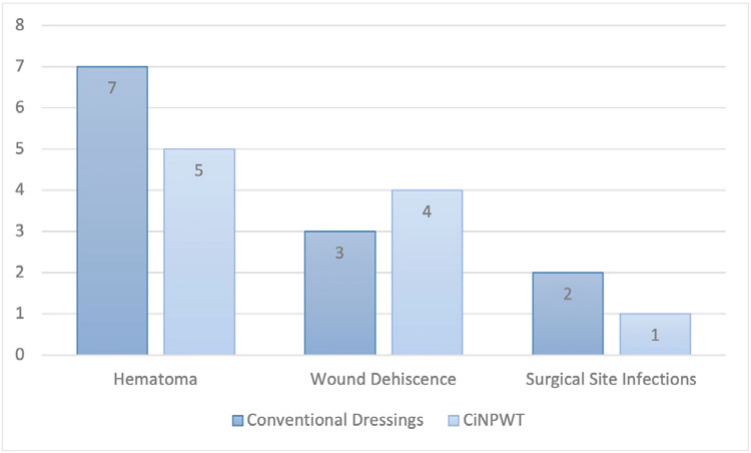
Comparison of Reasons for Returning to the Operating Room Within 30 Days.

Regarding hospital readmission and surgical revision rates within the first 30 days, there were no statistically significant differences between the ciNPWT and control groups (Table 4). In the group with conventional dressings, a total of 12 patients required one or more surgical revisions. Among these 12 patients, 7 underwent a single revision due to a hematoma, followed by skin suturing. Three patients were surgically treated for wound dehiscence with appropriate debridement, wound bed conditioning using a vacuum dressing and secondary suturing after 7 days. Two patients underwent two surgeries each due to post-operative SSI, involving surgical infection management, temporary closure of soft tissues with a vacuum dressing and subsequent second look surgery with secondary suturing (Figure 2). In the two cases with confirmed SSI, *Proteus mirabilis* and *Prevotella denticola* as well as *Escherichia coli* and *Enterococcus faecalis* were detected. A total of 18 operations were performed.

In the ciNPWT group, a total of ten patients returned to the operating room within 30 days. In five cases, the reason was post-operative hematoma formation requiring surgical evacuation and primary suturing. In four cases, the indication was wound dehiscence, leading to surgical necrosectomy, irrigation and secondary suturing. In one case, surgical infection management was performed with debridement, temporary vacuum dressing and a second look operation after 7 days with secondary suturing. *Serratia marcescens* was detected in the intraoperative samples (Figure 2). A total of 11 operations were performed. In addition, the duration of the second hospital stay was significantly shorter, averaging 5.8 days compared to 12.0 days, primarily because only one revision surgery was necessary in nine out of ten cases. In contrast, in the conventional dressing group, 4 out of 12 patients underwent two revisions and one patient required three revisions.

## Discussion

In the late 1990’s, the vacuum-assisted closure for wound control and treatment was first described for treating chronic oedema, increasing localised blood flow and building granulation tissue.^8^ Approximately 15 years later, the CiNPWT was introduced first for wounds with persistent secretion in the early post-operative care.^9^ In the last decades, numerous studies have compared the effectiveness of prophylactic CiNPWT to conventional dressings in joint replacement^10^ vascular surgery,^11^ gynaecology, spinal fusion,^12^ autologous breast reconstruction,^13^ inguinal lymph node resection^14^ or post-bariatric surgery.^5^ Most of the meta-analyses report a reduction in SSI and wound dehiscences.^10^, ^11^, ^12^^,^^15^

Post-bariatric surgery presents unique challenges and considerations due to significant weight loss and changes in body composition. Special attention needs to be paid to the management of excess skin, nutritional deficiencies and psychological adjustments to achieve optimal outcomes. Body contouring after massive weight loss introduces several possible complications. The most frequent events are wound dehiscence (29.0%), seroma (18.6%), scarring (14.9%) and infections (8.8%).^16^ In body contouring following bariatric surgery, there is a 37% greater risk of developing adverse surgical outcomes if the BMI is above 30 kg/m². In addition, a greater proportion of resected tissue correlates with an increased probability of complications.^17^ To mitigate these biases, we designed this study by comparing two groups matched based on the resection weight. In the analysis of baseline data, both groups exhibited no significant differences in terms of age, BMI, relative BMI reduction, cut-to-stitch time and pre-existing conditions except for psychological diseases (Table 2). This trial found no statistically significant difference among the two treatment modalities regarding primary or secondary endpoints except for the duration of the second hospital stay after revision surgery (Table 4). In addition to the previously mentioned hematomas, wound dehiscences and SSIs as indications for revision surgeries within the first 30 days, there was also an identical formation of seromas. The removal of all drainages also showed no significant difference between the two groups, so based on our data, we assume that ciNPWT has no influence on the formation of seromas in our patient cohort. The ciNPWT also had no influence on other post-operative complications such as post-operative bleeding or transfusions. One possible explanation for the non-significant differences in favour of ciNPWT could be an insufficient sample size. Contrastingly, a larger and retrospective sample size might not achieve the same balanced distribution of patient metrics.

According to Dragu and colleagues, ciNPWT wound dressings over the anterior and lateral trunk after post-bariatric abdominal dermolipectomy result in a substantial decline in exudate formation, early drain removal and consequent shorter hospital stay when compared to the conventional treatment group.^6^ In addition, Abatangelo and co-workers suggested that ciNPWT may decrease the rate of minor local complications in post-bariatric patients who wished to undergo body contouring procedures.^2^ In a third publication on abdominoplasties and ciNPWT, the interventional group demonstrated a significantly reduced amount of fluid drainage and the time before the removal of both drains was decreased.^18^ However, the largest cohort receiving ciNPWT after post-bariatric abdominoplasty among these three studies included 23 patients. Thus, none of the three mentioned studies can be considered to have a meaningful cohort size. To our knowledge, no other studies on the benefits of CiNPWT in post-bariatric surgery exist. The data presented in this study are thus based on the largest cohort to date. Moreover, in a comparable examination of abdominoplasties during deep inferior epigastric perforator flap breast reconstructions, no significant change in complications was observed between the two groups similar to our results.^19^

In other surgical settings, ciNPWT is frequently cited as a valuable additional treatment for decreasing the incidence of surgical site complications. Meta-analyses of randomised controlled trials in total hip or knee arthroplasty have consistently shown that ciNPWT reduces the rates of SSI, especially in cases of revision arthroplasty and among high-risk patients.^20^ Additionally, in cardiothoracic surgery, single-use ciNPWT has been proven to be cost-effective, contributing to a reduction in sternal wound infections and a shorter length of hospital stay in high-risk patients predisposed to such infections.^21^ However, devices and operation methods vary highly heterogeneous among patient cohorts and the results are not universally generalizable. Other case-control studies, for example from orthopaedics, have mixed results and do not show an advantage of using ciNPWT with respect to the overall wound complication rate.^19^ Further, Newman and colleges demonstrated equal readmission rates between patients treated with ciNPWT and controls in a high-risk cohort of patients who underwent total knee arthroplasty or total hip arthroplasty.^20^ Other working groups claimed no significant difference in deep SSI rate between the ciNPWT and control groups but the overall wound complication rate was significantly lower in cases where ciNPWT had been used.^21^ In gynaecology, ciNPWT was compared with standard dressings in 970 patients after a caesarean section. CiNPWT did not have a statistically significant effect on reducing the rates of wound dehiscence and hematoma formation or on increasing long-term satisfaction with the appearance of scar tissue after caesarean section.^22^ A meta-analysis published in 2022 on ciNPWT use in spine surgery suggested that ciNPWT could effectively reduce post-operative SSI, but it had no significant benefit on reducing the incidence of wound dehiscence, overall wound complications or readmissions.^23^ CiNPWT also presents several disadvantages, including higher costs, increased skin complications (e.g. blisters), limited long-term outcome evidence and specific contraindications that limit its use in certain patient populations (tumours, exposed nerves or vessels and persistent necrosis).^24^ Therefore, it must be assumed that only certain patient groups with an already increased risk of SSI benefit from ciNPWT use. Based on the recent literature, ciNPWT is not always superior to conventional methods and further research is needed to identify the conditions under which ciNPWT might be more beneficial.

Several systems for the application of ciNPWT exist, such as PICO® single-use portable negative pressure wound therapy system (Smith & Nephew plc, Watford, England, UK) or PREVENA™ Incision Management System (KCI, San Antonio, TX, USA). There is an ongoing debate regarding the advantages and disadvantages of these systems.^25^ Overall, the devices—whether with foam, gauze or an embossed membrane—performed equally well.^26^ In our retrospective case-control study, we used the standard vacuum system by KCI (KCI, San Antonio, TX, USA). As this system is not intended for outpatient use, patients remained in our care at the hospital until the completion of the epicutaneous vacuum therapy. Consequently, the length hospital of stay was longer in our cases compared to similar studies using the Pico® or PREVENA™ systems (Table 1). Up to now, no trials have compared the mentioned devices and their secondary outcome parameters, such as wound dehiscence or SSI. Future studies should compare the systems and their efficiency against each other.

Special challenges arise in post-bariatric surgery due to the higher risk of nutritional deficiencies. Within our own patient group, we identified deficiencies in iron, zinc and vitamin B1 (Table 3). Levels of ferritin, folic acid and vitamin B12 were normal, and there was evidence of hypersubstitution of vitamin B6. Notably, all patients were adequately supplemented according to the guideline recommendations. A protein deficiency was ruled out in our cohort, which demonstrated normal serum albumin levels (Table 3). The respective values for the complete blood count, coagulation profile, serum electrolytes and HbA1c revealed normal parameters in both groups. No further significant differences between the groups in the preoperative standard laboratory tests were detected.

The strengths of our study lie in the large patient cohort, two comparable groups, robust statistics and exclusion of potential confounding factors in patient characteristics or laboratory parameters. However, this study has several limitations. First, its retrospective design may introduce bias. We aimed at mitigating this by mandating data retrieval and collection to a professional Data Integration Centre. Nonetheless, parameters such as drainage flow rates or vacuum pump flow rates cannot be accurately determined in retrospect. Other factors influencing wound healing such as the extensive use of electrocautery, a traumatic surgical method, high tension on the wound edges or complete exposure of the abdominal fascia cannot be quantified retrospectively and may distort the results as confounding variables. Owing to insufficiently documented data, the retrospective assessment of scar quality and its verification using a validated tool such as the Vancouver Scar Scale was not possible. Therefore, no definitive statements can be made regarding the scar quality after ciNPWT. Additionally, the observed differences in patient and surgical characteristics that could potentially act as confounders were noted, but not statistically controlled for as additional parameters in the matching process. Although we present data based on the largest sample size in post-bariatric patients, it might have been insufficient to detect the effect of ciNPWT. Under economic considerations, additional costs occurred by prolonged hospital stay or the materials used remain unaccounted for.

To address these limitations, future research should involve prospective randomised controlled trials to evaluate the prophylactic effect of ciNPWT in abdominoplasty after massive weight loss. Additionally, endpoints such as cost-effectiveness, length of hospital stay, scar quality and patient satisfaction should ideally be examined.

## Conclusion

We observed no statistical difference in the incidence of minor or major complications when comparing conventional wound dressings to ciNPWT used for 7 days in abdominoplasties after massive weight loss. Furthermore, no statistical difference was demonstrated in the rates of return to the hospital or return to the operating room within the first 30 days post-operatively. In fact, the use of ciNPWT did not affect the rates of readmission or revision. The only discernible difference was a shortened hospital stay upon readmission observed in the intervention group with ciNPWT.

## Role of the funding source

The APC was funded by the Open Access Publishing Fund of the University of Leipzig.

## Ethical approval

This study was approved by the ethical committee of the University of Leipzig with permission number: 088/23-ek, 04.04.2023.

## Declaration of competing interest

None.
